# Microarray Analysis of Differentially Expressed Genes in Peripheral Blood of Postpartum Women with Gestational Diabetes Mellitus and Type 2 Diabetes

**DOI:** 10.3390/life15081270

**Published:** 2025-08-11

**Authors:** Samar Sultan

**Affiliations:** 1 Medical Laboratory Sciences, Faculty of Applied Medical Sciences, King Abdulaziz University, Jeddah 21589, Saudi Arabia; sasultan@kau.edu.sa; Tel.: +966-126400000; 2 Regenerative Medicine Unit, King Fahad Medical Research Centre, King Abdulaziz University, Jeddah 21589, Saudi Arabia

**Keywords:** gestational diabetes, type 2 diabetes, microarray analysis, mRNA expression

## Abstract

The etiology of women with gestational diabetes mellitus (GDM) and a greater risk of developing type 2 diabetes (T2D) after delivery remains unknown. This study aimed to investigate the global gene expression in four postpartum women with previous GDM (pGDM), three with T2D, and three with a history of normoglycemic pregnancy (controls). Total RNA was extracted from whole blood between March and May 2020. Global mRNA expression was determined using an Affymetrix Human Gene 2.0 ST Array. The expression of the selected focused genes was validated by RT-PCR. The microarray revealed 140 transcripts (*p* < 0.05, fold change cut-off ≥ 2) in patients with pGDM compared to controls. We identified 583 gene-altered transcripts between patients with T2D and controls. Interestingly, 60 transcripts had genes shared by pGDM or T2D versus the controls. The selected upregulated genes involved in inflammatory response, glycosylation, and death-like domains, according to the functional network analysis of pGDM (*TNFAIP6*, *PDK3*) and T2D (*MMP9* and *CARD6*), showed similar trends to those obtained via microarray. Thus, these differentially expressed genes and their corresponding network and pathway analyses in women with pGDM and T2D offer valuable insights into the possible biological mechanisms of the progression of GDM to T2D.

## 1. Introduction

The etiology of diabetes is complicated and multifactorial, and alterations in gene transcripts may play a role in the pathogenesis of this metabolic disease. Type 2 diabetes (T2D), the most common type of diabetes, is caused by decreased insulin sensitivity and resistance [1]. Gestational diabetes mellitus (GDM) is the most common metabolic disturbance in pregnancy, occurs due to impaired glucose tolerance, and usually disappears following delivery [2]; however, up to 35% of cases progress to T2D within the first two months following delivery [3]. This is supported by long-term follow-up studies showing that women with previous GDM (pGDM) are at a high risk of developing T2D and associated cardiovascular diseases (CVDs) later in life [4,5]. The mechanisms of progression to T2D are not thoroughly understood and could be attributed to risk factors such as family history, obesity, and the need for insulin treatment [6,7]. Women with a history of GDM develop insulin resistance, dyslipidemia, and hypertension, which can lead to T2D and CVD [8,9]. Moreover, a recent study by Van et al. showed that notable inflammation and elevated triglyceride levels occurred shortly after delivery and were more pronounced in women with progression to overt T2D [10].

The pathophysiology of GDM is similar to that of T2D. GDM develops in pregnant women during late pregnancy as a result of an increase in insulin resistance and reduced insulin sensitivity compared to those in non-pregnant women [11]. This increase in insulin resistance leading to GDM is due to pancreatic beta cell insufficiency [12].

Several lines of evidence support the involvement of genetic components in the progression of GDM to T2D. A previous study showed a 2.3-fold higher risk of GDM in women with any parental history of diabetes compared to women with non-diabetic parents [13]. The risk of GDM increased 8.4-fold in women with diabetic siblings than in women without diabetic siblings [13].

The concept of glycemic memory or metabolic memory, which occurs when human cells are exposed to sustained elevated blood glucose levels, has recently received increasing attention. Sustained exposure to hyperglycemia can epigenetically alter gene expression profiles, and this effect persists even after therapeutic hyperglycemic control is therapeutically achieved [14]. However, the underlying mechanisms are not fully understood. Interestingly, it has been shown that oxidative-stress-induced metabolic memory is responsible for diabetes progressing to complications such as retinopathy and CVDs [15,16].

Changes in gene transcript expression in women with pGDM may also be implicated in the development of T2D [17]. In our previous study, we showed alterations in the antioxidant enzymes superoxide dismutase and sirtuin transcripts and protein in women with both pGDM and T2D, which may be related to oxidative stress [17]. To the best of our knowledge, there is no known global gene expression profile in the blood of women with pGDM. Therefore, the aim of this study was to identify changes in gene expression in peripheral blood at one day postpartum in women with GDM and T2D compared to non-diabetic women (controls) using microarray analysis and to validate the selected genes via real-time polymerase chain reaction (RT-PCR).

## 2. Materials and Methods

### 2.1. Samples

Peripheral whole blood samples were collected one day postpartum from four women with pGDM (pGDM group), three healthy women (control group), and three women with T2D (T2D group) after a full-term pregnancy, normal delivery, and matched age between March and May 2020. One day postpartum is the typical amount of time postpartum pregnant mothers are allowed to stay at the hospital after normal delivery. The subjects in the control group were non-smokers, did not have a history of T2D, and did not have hypertension. Individuals in the T2D group received insulin along with additional medications, including metformin. Mothers with GDM or T2D were selected based on the recommendations of the American Diabetes Association [18]. Mothers with GDM had no other complications, such as preeclampsia or hypertension, and they were on standardized diets. All participants were informed about the objectives of the research; informed written consent was obtained before inclusion; and the study was performed in accordance with the Declaration of Helsinki and approved by the Ethics Committee of the Research and Studies Department, Directorate of Health Affairs, Jeddah (IRB registration number: H-02-J-002; approved on 29 January 2020). The donors’ physical examination results, such as weight, height, and BMI, were recorded. Biochemical analyses were performed using the patients’ medical records.

### 2.2. RNA Extraction and Microarray Processing

Total RNA (250 ng/sample) was isolated from 2.5 mL of whole blood using the PAXgene Blood RNA System (Qiagen, Manchester, UK) following the manufacturer’s instructions. The RNA concentration and purity were measured spectrophotometrically using a NanoDrop spectrophotometer (Nano-Drop Technologies, Wilmington, DE, USA). The quality of RNA was additionally confirmed through analysis with an Agilent bioanalyzer (Agilent Technologies, Boeblingen, Germany) prior to the hybridization of the microarray chip. The GeneChip^TM^ Whole Transcript PLUS kit (Affymetrix, Santa Clara, CA, USA) was used to screen for gene expression in patients with pGDM and T2D according to the manufacturer’s instructions. Extracted RNA was amplified and transcribed into cRNAs. The obtained cRNAs were then hybridized to Human Gene 2.0 ST arrays (Affymetrix, Santa Clara, CA, USA), which measured the signals for >30,000 coding transcripts and >11,000 long intergenic non-coding transcripts. The Affymetrix GeneChip Hybridization, Wash, and Stain Kit was used for hybridization. Sample hybridization to the GeneChip microarray (performed in triplicate) and image scanning were performed according to the manufacturer’s instructions.

### 2.3. Microarray Enrichment Analysis

Microarray expression data were imported into Bioconductor version 3.7. The data were normalized to minimize the effects of systematic non-biological variations. The analysis was focused on genes with a fold change ≥ 2 and *p*-value < 0.05 in pGDM and T2D relative to the controls. For subsequent functional clustering of the differentially expressed genes, they were enriched into the Gene Ontology (GO) terms cellular component, biological process, and molecular function using the GO chart feature of the Database for Annotation, Visualization, and Integrated Discovery (DAVID), and the results were ranked based on enrichment scores.

Data sets that include gene identifiers along with their corresponding expression values (fold change) from the microarray analysis were uploaded into the IPA software (build version 463341M, Ingenuity^®^ Systems, www.ingenuity.com, accessed on 20 March 2018). Each gene identifier was matched to its corresponding gene entity in the Ingenuity Pathways Knowledge Base. We utilized data sources from Ingenuity expert insights and employed the ‘Core Analysis’ function to interpret the data in terms of biological processes, pathways, and networks. Differentially expressed gene identifiers were established as value parameters for analysis, revealing the relationship between alterations in gene expression and associated changes in biofunctions under the following categories: Molecular and Cellular Functions, Physiological System Development and Function, and Disease and Disorders. Genes that were differentially expressed with a *p*-value of less than 0.05 were overlaid onto global molecular networks created from the information available in the IPA knowledge base. These networks were then algorithmically generated based on their connectivity. The networks were ‘named’ according to their most prevalent functional group(s) that were identified. Canonical pathway (CP) analysis pinpointed function-specific genes that were significantly present within the networks.

### 2.4. Validation of Selected Genes Using RT-PCR

Total RNA (1 µg) was used to synthesize cDNA using an ImProm-II Reverse Transcription System kit (Promega, Madison, WI, UK), following the manufacturer’s protocol. Primers for the target genes were designed using the Primer3Primer software version 6.2.0. β-actin was used to normalize mRNA levels. Sequences of the target primers and β-actin are listed in Table 1.

Quantitative RT-PCR was performed in duplicate using a QuantiTect SYBR Green PCR kit (Qiagen, Manchester, UK) on an iCycler iQ Real-Time PCR Detection System (Applied Biosystems, Cheshire, UK) according to the manufacturer’s instructions. Reactions were run according to the following profile: 10 min denaturing at 95 °C; and 40 cycles of denaturing at 95 °C for 15 s, annealing at 63 °C for 10 s, and extension at 72 °C for 20 s. Data analysis was performed using the Rest 2009 software version 2.0.13 [19].

### 2.5. Statistical Analysis

Unpaired data were analyzed using Student’s *t*-test. The results are presented as mean ± SEM. Differences were considered statistically significant at *p* < 0.05.

## 3. Results

The basic clinical characteristics of the study participants are presented in Table 2.

Random plasma glucose levels were significantly higher in women with T2D than in the controls (*p* = 0.01). However, the other parameters did not differ among the pGDM, T2D, and control groups.

**Figure 1:**
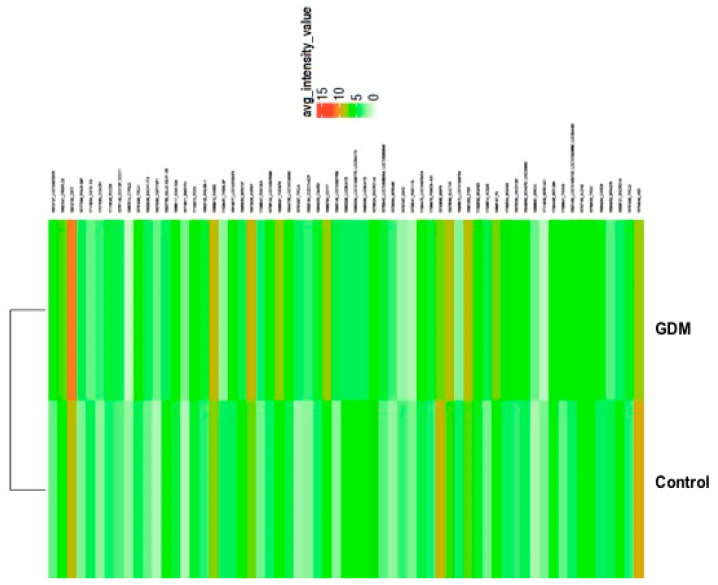
Heatmap and hierarchical cluster analysis of differentially expressed genes. The heatmap shows the relative induction or repression of genes in gestational diabetes mellitus (GDM) and controls. Green indicates higher expression; red indicates reduced mRNA expression; light green indicates no changes.

The isolated RNA purity was assessed using a 260/280 ratio and was approximately 2.0. mRNA analyses were performed by comparing the two groups with the controls, i.e., pGDM versus controls and T2D versus controls. We identified 140 transcripts that were differentially expressed between the pGDM and control groups (*p*-value < 0.05, fold change cut-off ≥ 2). A total of 106 genes were upregulated, and 34 genes were downregulated in the pGDM group compared to the controls, as indicated by the heatmap (Figure 1, Table 3).

The most upregulated genes in pGDM included maltase-glucoamylase 2, while the most significantly downregulated genes in pGDM were small nucleolar RNA C/D box 1B. Functional annotation cluster analysis was performed using DAVID, which showed that these 140 differentially expressed genes were involved in the immunoglobulin complex, glycosylation, and proteolysis (Table 4).

Additionally, an examination of the genes mentioned earlier (140 gene transcripts) uncovered four notable genetic networks (*p* < 0.05, enrichment score ≥ 2) in pGDM using IPA, as detailed in Table 5.

**Figure 2:**
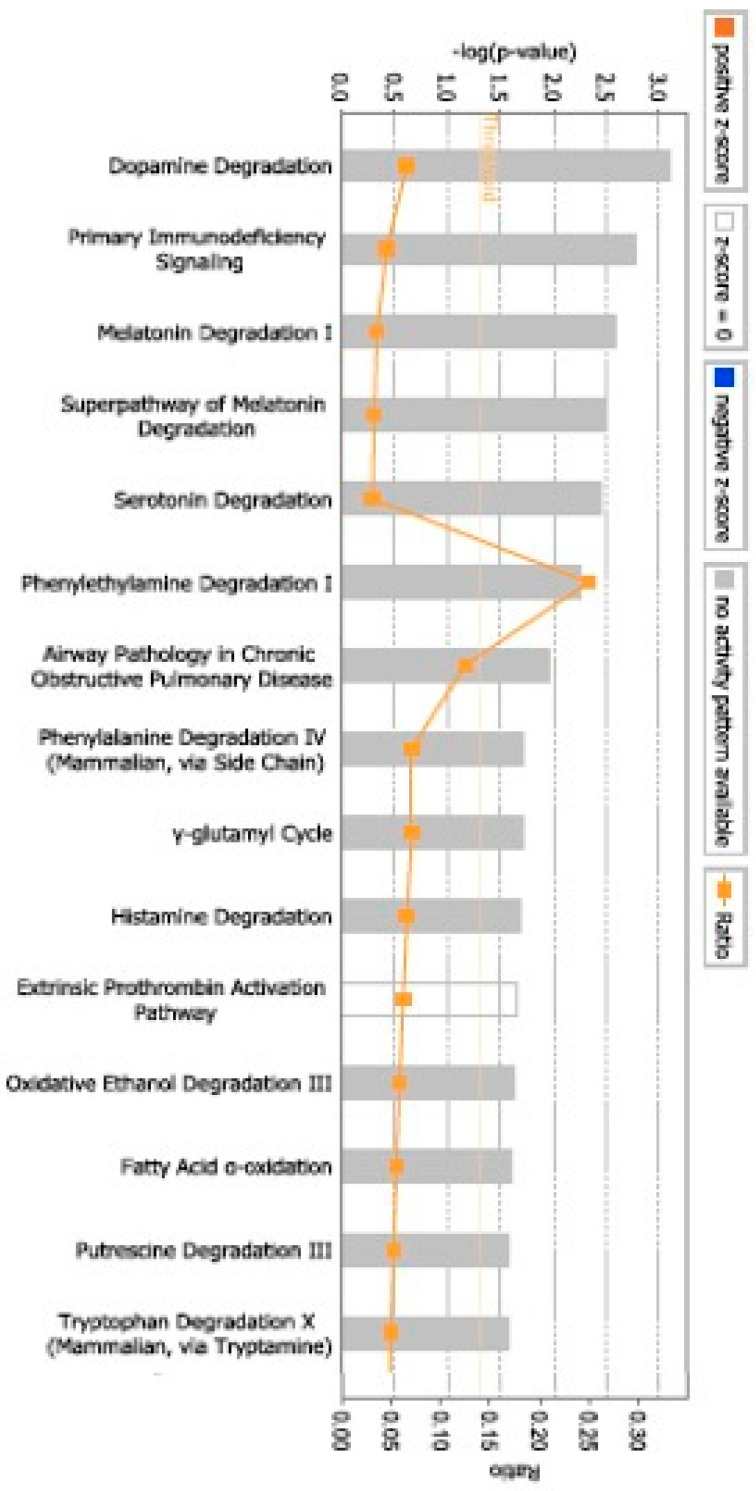
Canonical pathways (CPs) associated with pGDM subjects. CPs that were identified as the most statistically significant in the IPA core analysis are presented in accordance with their *p*-values (−Log). The threshold line represents a *p*-value of 0.05.

The highest-scoring networks indicated associations with diseases and disorders that mainly comprised (number of molecules) Cancer (13), Cardiovascular Disease (5), Connective Tissue Disorders (2), Dermatological Disease and Conditions (6), and Developmental Disorders (2). Genes within the Molecular and Cellular Functions network included Drug Metabolism (3), Carbohydrate Metabolism (1), Cell-to-Cell Signaling and Interaction (6), Cellular Assembly and Organization (5), and Energy Production (1). In the Physiological System Development and Functions network, the significant functions were Embryonic Development (4), Organ Development (4), Organ Morphology (1), Organismal Development (5), and Organismal Functions (1). Our IPA analysis indicates that multiple significant CPs are impacted by pGDM, i.e., Dopamine Degradation (Figure 2).

**Figure 3:**
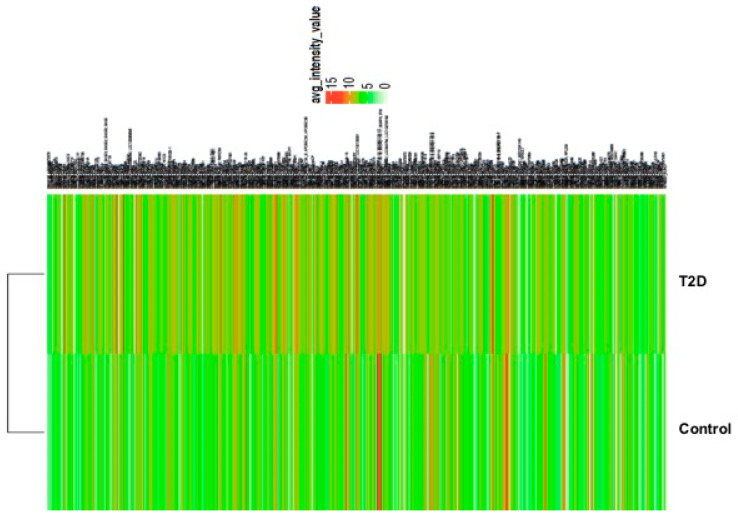
Heatmap and hierarchical cluster analysis of differentially expressed genes and validation of indicated selected mRNA by qRT-PCR. The heatmap shows the relative induction or repression of genes in T2D and controls. Green color indicates higher expression; red color indicates reduced expression of mRNA; light green indicates no changes.

In patients with T2D, 583 gene transcripts were significantly altered in comparison with controls (*p* < 0.05). Of these, 490 showed an increase, and 93 displayed a decrease in transcript levels, as depicted by the heatmap (Figure 3, Table 6).

The most upregulated genes in T2D included transmembrane and tetratricopeptide repeat containing 1 (TMTC1). The most significantly downregulated genes were interferon-induced protein with tetratricopeptide repeat 1B (IFIT1B). The functional annotation cluster showed that these 583 differentially expressed genes were involved in immune response, glycosylation, and death-like domains (Table 7).

**Figure 4:**
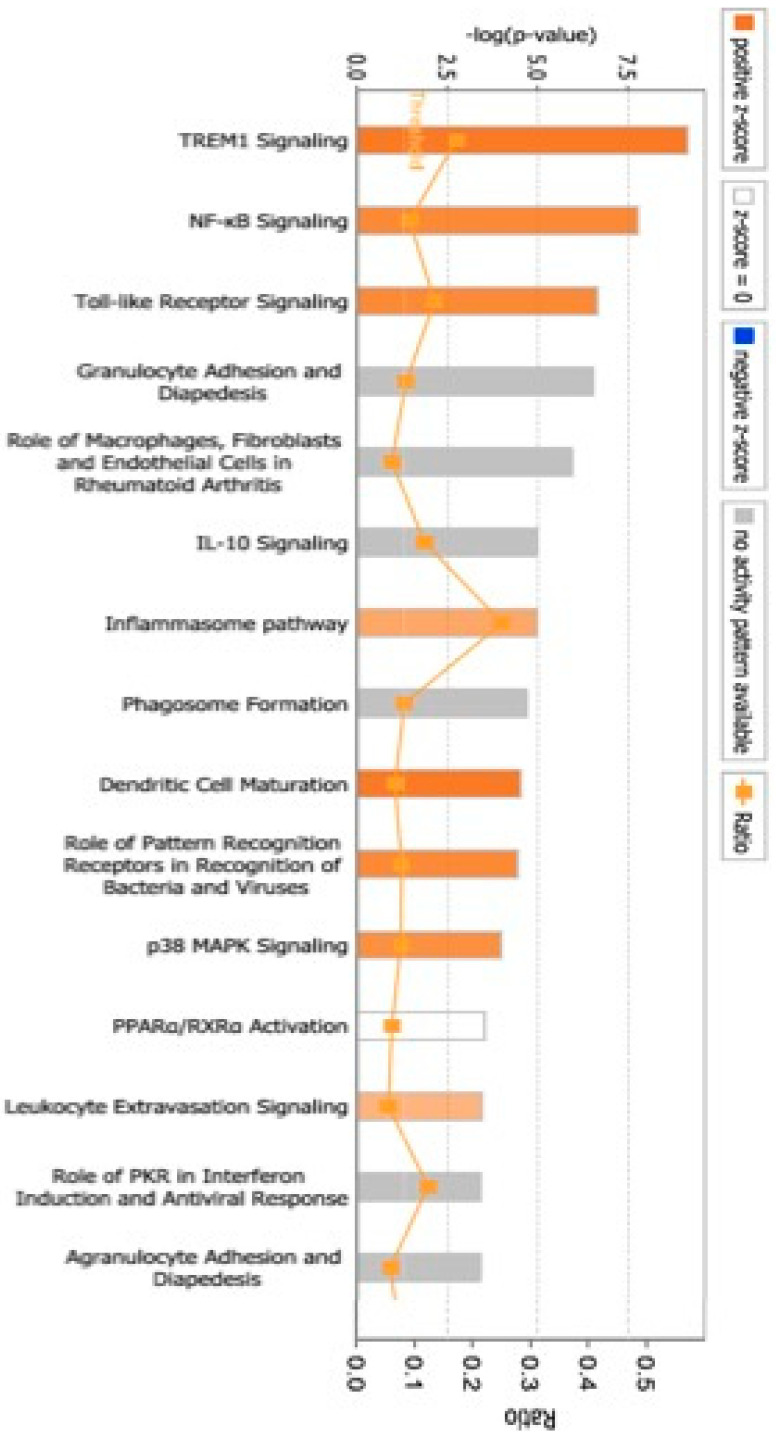
CPs associated with T2D subjects. CPs that were identified as the most statistically significant in the IPA core analysis are presented in accordance with their *p*-values (−Log). The threshold line represents a *p*-value of 0.05.

Furthermore, a review of the previously mentioned genes (583 gene transcripts) revealed five significant genetic networks (*p* < 0.05, score ≥ 23) in T2D through the use of IPA, as outlined in Table 8. The networks with the highest scores suggested associations with diseases and disorders that primarily included (number of molecules) Connective Tissue Disorders (66), Immunological Disease (120), Inflammatory Disease (90), Inflammatory Response (95), and Organismal Injury and Abnormalities (282). Genes located within the Molecular and Cellular Functions network included Cell Death and Survival (103), Cell-To-Cell Signaling and Interaction (72), Free Radical Scavenging (20), Cellular Movement (68), and Post-Translational Modification (30). In the Physiological System Development and Functions network, the important functions were Hematological System Development and Function (51), Immune Cell Trafficking (38), Digestive System Development and Function (14), Hepatic System Development and Function (14), and Organ Development (19). Our IPA analysis reveals that several important CPs are affected by T2D, specifically TREM1 Signaling (Figure 4).

**Figure 5:**
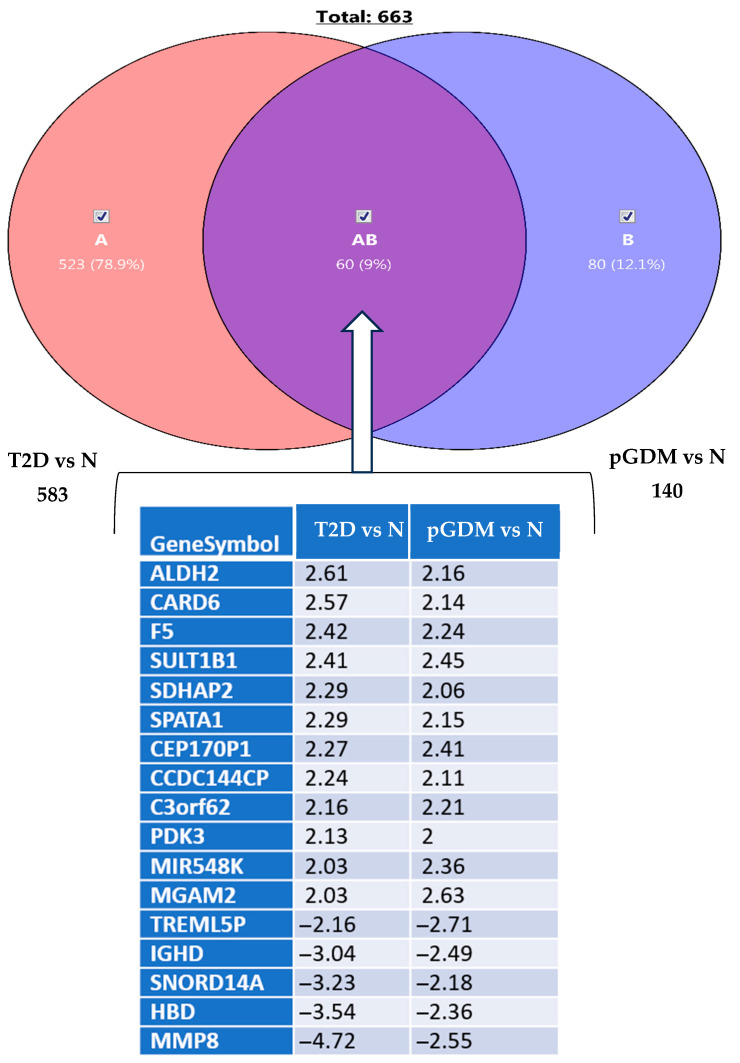
Venn diagrams showing the total number of shared genes between pGDM or T2D (A and B, respectively) and controls (N). Sixty differentially expressed transcripts were shared by pGDM and T2D (AB), of which seventeen are displayed along with their gene symbols and fold changes (*p* < 0.05, fold change cut-off ≥ 2).

Furthermore, 60 differentially expressed transcripts (*p* < 0.05, fold change cut-off ≥ 2) that were shared by pGDM and T2D are presented in Figure 5.

**Figure 6:**
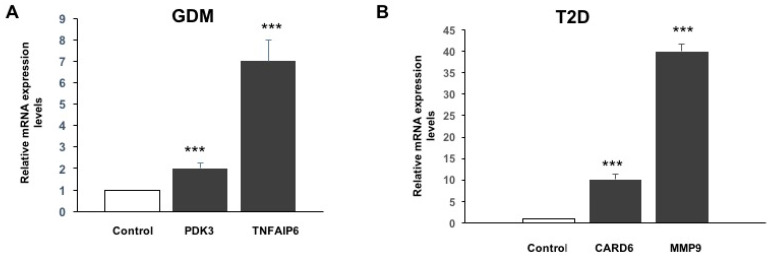
Validation of the indicated selected mRNAs by qRT-PCR. Total RNA isolated from peripheral blood mononuclear cells of (**A**) GDM and (**B**) T2D and the indicated selected target genes tested using qRT-PCR. The results are depicted as fold change over a housekeeping gene (b-actin). The results are expressed as mean ± SEM (*n* = 3–4). *** *p* < 0.0001 versus controls.

According to the functional network analyses (DAVID and IPA), we focused on the upregulated genes involved in inflammatory response, glycosylation, and death-like domains, as these could play a role in the pathogenesis of diabetes and its associated complications (Table 4, Table 5, Table 7, and Table 8). Combining the differentially expressed gene lists of pGDM versus controls (Table 3) and the gene clusters in Table 4 and Table 5, we extracted two key genes (*TNFAIP6* and *PDK3*) for forward RT-PCR validation. Similarly, *MMP9* and *CARD6* were selected when we compared the differentially expressed gene lists of patients with T2D versus controls, as shown in Table 6, Table 7 and Table 8. For each of the four genes (*TNFAIP6*, *PDK3*, *MMP9*, and *CARD6*), the mRNA levels showed trends consistent with those observed in the microarray analysis. The expression of *TNFAIP6* and *PDK3* increased significantly, approximately 7- (*p* = 0.0001) and 2-fold (*p* = 0.0001), respectively, in the pGDM group compared to the controls (Figure 3). The mRNA levels of MMP9 and CARD6 were significantly upregulated, approximately 10.1- (*p* = 0.0001) and 40-fold (*p* = 0.0001), respectively, in patients with T2D compared to the controls (Figure 6).

## 4. Discussion

Women with pGDM are at a higher risk of developing T2D later in life [20], and the fundamental pathophysiological mechanisms that contribute to this remain incompletely understood. The role of inflammation in the onset of GDM has become a central focus in recent research [21]. Modified glycosylation patterns could act as potential biomarkers of the pathophysiology related to diabetes and could be used for therapeutic targeting [22]. Apoptosis plays an important role in the pathogenesis of diabetes, as hyperglycemia induces reactive oxygen species (ROS). This leads to considerable cellular damage and to a point of no return in apoptosis when insufficient cytoprotective and ROS scavenging molecules, such as catalase and superoxide dismutase, are available [23]. In this study, our microarray results revealed 140 differentially expressed transcripts in pGDM compared to the controls. A total of 583 differentially expressed transcripts were identified in patients with T2D compared to the controls. There were 60 shared genes between T2D versus controls and GDM versus controls, and this could imply possible disease progression to T2D. Of these differentially expressed genes, *PDK3* and *TNFAIP6* in pGDM and *MMP9* and *CARD6* in T2D were selected, as they are involved in inflammatory response, glycosylation, and death-like domains based on DAVID and IPA function analyses, which may be involved in the pathological progression to T2D. RT-PCR validation showed that these genes were upregulated, which confirmed the microarray results.

In glucose metabolism, an irreversible mitochondrial oxidative decarboxylation reaction of pyruvate to acetyl-CoA is catalyzed by the action of both enzymes’ pyruvate dehydrogenase kinases (PDKs) 1–4 and pyruvate dehydrogenase phosphatases 1–2 [24]. Our results are the first to show that *PDK3* expression is increased in women with pGDM compared to the controls. To the best of our knowledge, no study has investigated *PDK3* levels in patients with GDM or diabetes. However, upregulation of *PDK4* and *PDK1* expression has been linked to obesity [25] and diabetes [26], respectively. Notably, because *PDK3* is shared between pGDM and T2D, it could serve as a biomarker for diabetes development.

TNFAIP6 or TSG6 is a secretory protein produced by inflammatory cells in response to inflammatory stimuli, such as TNF [27]. It acts as an anti-inflammatory agent by upregulating cytokines, such as IL-4 and IL-10, and preventing the migration of neutrophils [28]. Our previous study showed that proatherogenic inflammatory cytokines increased in GDM-derived fetal endothelial cells compared to those in control non-diabetic fetal endothelial cells after several passages of culture under normal glucose conditions, supporting the concept of glycemic memory or programming [29,30]. Subclinical inflammation has been observed in women with a history of GDM [31], which could at least be partially explained by the observed increase in *TSG6* in patients with pGDM compared with the controls. However, this hypothesis warrants further investigation. Many studies have reported an association of subclinical inflammatory markers with increased levels of IL-6, C-reactive protein, plasminogen activator inhibitor-1, and the future development of T2D and CVD [32,33]. Interestingly, IPA determined that TNFAIP6 is a molecule involved in the Carbohydrate Metabolism as well as Cardiovascular Disease networks. To our knowledge, no study has investigated the expression of *TSG6* in patients with GDM or diabetes, and the overexpression of *TSG6* suggests its possible role in the later development of diabetes.

MMPs are zinc-dependent endopeptidases consisting of 25 members with similar structures and functions that are genetically different [34]. This family exerts catalytic activity against various extracellular matrix components, including proteoglycans, laminins, and collagens [35]. Under normal conditions, their expression levels and activity are low, but they increase under pathological conditions, such as CVD [36]. We found an increase in *MMP9* levels in the T2D group compared with those in the control group. Consistent with previous studies, *MMP9* was overexpressed in patients with diabetes [37] and streptozotocin-induced diabetic rats [38]. In addition, the in vitro exposure of endothelial cells to elevated glucose levels causes an increase in *MMP9* [39]. Increased ROS and nitric oxide synthase have been shown to enhance the activity of MMP9 in placental tissue from patients with GDM [40]. We did not test the reduced expression of MMP8 in T2D using RT-PCR to rule out errors. Therefore, the mechanisms behind the increased MMP9 expression with concurrent reduction in MMP8 expression in women with T2D are unclear, and this merits further investigation. These results suggest that in the diabetes context, high glucose, oxidative stress, and insulin resistance could modify the expression and activity of *MMPs*, and these could serve as risk markers for diabetes.

Apoptosis is a biochemical process that leads to specific morphological changes and eventual cell death. The pro-apoptotic protein CARD6 has been suggested to trigger nuclear factor kappa beta (NFκB) activation and regulates the function of RIP kinase family members [41]. An oxidative-stress-induced inflammatory secretion such as NF-κB has been shown to play a role in the development of insulin resistance and diabetes [42]. In the current study, CARD6 mRNA was overexpressed in the T2D group compared to the controls and appeared to be shared between the pGDM and T2D groups, suggesting its role in the pathogenesis of diabetes. Consistent with another study, *CARD6* expression was increased in retinal cells isolated from streptozotocin-induced diabetic rats and in retinal pigment epithelium cells, and it was involved in cell apoptosis and oxidative stress [43,44]. To the best of our knowledge, no study has investigated the expression of *CARD6* in GDM or pGDM. Further study is required to characterize the role of CARD6-induced NF-κB in GDM and pGDM.

The main limitation of this study is its small sample size due to budget constraints. Despite this, our results are supported by strong statistical results and highlight altered gene transcripts that could be used as biomarkers for diabetes, and these results are strengthened through multiple experiments. Similar studies in the literature include few samples [45,46]. To the best of our knowledge, there are no studies regarding global gene expression in pGDM to explore the possible biomarkers of T2D progression. Thus, our study can be considered a pilot for future research with larger numbers of samples and higher budgets to validate our results and protect postpartum mothers with GDM from progression to T2D. We also did not have information regarding the ethnicity and lifestyle of the individuals, which may influence gene expression. We did not directly test the impact of insulin and metformin treatment in T2D patients. However, this is worthy of further investigation. The functional validation (e.g., knockdown, pathway modulation) of the selected gene transcripts merits future investigation.

## 5. Conclusions

This study provides insights into differentially expressed genes and their associated biological networks and pathways in pGDM and T2D. The potential genetic biomarkers we obtained (*PDK3*, *NFAIP6*, *MMP9*, *CARD6*) could be implicated in the pathogenesis of GDM and progression to T2D. It is essential to validate these biomarkers on larger and more diverse populations to evaluate their predictive value; afterward, they could be utilized to screen GDM individuals to predict T2D following GDM pregnancies. The integration of data from multiple studies is necessary to identify the target biomarkers associated with pGDM progression.

## Figures and Tables

**Table 1:** Primer sequences.

Primers	Sequences
PDK3	Forward: 5′-GAGCAATCCCAGCAGTGAAC-3′ Reverse: 5′-ATAACTGTGATGCCACGCTC-3′
TNFAIP6	Forward: 5′-GCTGGATGGATGGCTAAGGG-3′ Reverse: 5′-CCTTTGCGTGTGGGTTGTAG-3′
MMP9	Forward: 5′-GGTGATTGACGACGCCTTTG-3′ Reverse: 5′-GGACCACAACTCGTCATCGT-3′
CARD6	Forward: 5′-CGAGAGTACTCCCTCAGAGAT-3′ Reverse: 5′-GCCCCCATAGATTGAGGAGG-3′
β-actin	Forward: 5′-AGCGGGAAATCGTGCGTGAC-3′ Reverse: 5′-CGGACTCGTCATACTCCTGCT-3′

**Table 2:** Donors’ clinical characteristics.

Mothers’ Status	Controls (*n* = 3)	pGDM (*n* = 4)	T2D (*n* = 3)	*P* (pGDM vs. Controls/T2D vs. Controls)
Age (years)	28.6 ± 0.9	30 ± 1	31.7 ± 1.6	0.29/0.2
BMI	28.8 ± 3	30 ± 1.3	31.3 ± 3.9	0.37/0.32
HbA1c (%) RPG (mM) 2h OGTT	- 4 ± 0.3 -	6.1 ± 0.1 5.3 ± 0.9 9.9 ± 1	6.3 ± 0.5 5.5 ± 0.2 -	- 0.28/0.01 * -

RPG, random plasma glucose; BMI, body mass index; HbA1c, glycosylated hemoglobin; OGTT, oral glucose tolerance test. Data expressed as mean ± SEM. * *p*-value < 0.05.

**Table 3:** List of selected differentially upregulated and downregulated genes in previous GDM (pGDM) versus controls.

Gene Symbol	Fold Change	*p*-Value	Gene Name
MGAM2	4.7	0.0343	maltase-glucoamylase 2 (putative)
LOC105372578	4.36	7.38 × 10^−7^	uncharacterized LOC105372578
TRGV5	4.24	0.0414	T-cell receptor gamma variable 5
LOC100507639	3.94	0.0147	uncharacterized LOC100507639
TRDJ1	3.86	0.0019	T-cell receptor delta joining 1
TRDJ4	3.57	0.0095	T-cell receptor delta joining 4
CST7	3.55	8.22 × 10^−5^	cystatin F (leukocystatin)
CD177	3.54	0.0129	CD177 molecule
LOC102723373	3.35	0.0051	uncharacterized LOC102723373
HCG26	3.12	0.0326	HLA complex group 26 (non-protein-coding)
LINC01061	2.96	0.0353	long intergenic non-protein-coding RNA 1061
RNU6-59P	2.94	0.0004	RNA, U6 small nuclear 59, pseudogene
KIAA1324	2.86	0.0025	KIAA1324
ANPEP	2.75	0.0059	alanyl (membrane) aminopeptidase
TRDJ2	2.71	0.0493	T-cell receptor delta joining 2
RNU5B-1	2.64	0.0031	RNA, U5B small nuclear 1
SULT1B1	2.6	0.0237	sulfotransferase family 1B member 1
CEP170P1	2.57	0.0024	centrosomal protein 170 kDa pseudogene 1
TNFAIP6	2.51	0.0088	tumor necrosis factor, alpha-induced protein 6
BACH1-IT2	2.46	0.002	BACH1 intronic transcript 2
MIR548K	2.45	0.0159	microRNA 548k
PSMD5-AS1	2.41	0.0203	PSMD5 antisense RNA 1 (head-to-head)
DYSF	2.38	0.025	dysferlin
LOC105379818	2.37	0.0199	uncharacterized LOC105379818
F5	2.36	0.0333	coagulation factor V (proaccelerin, labile factor)
CRISPLD2	2.35	4.47 × 10^−5^	cysteine-rich secretory protein LCCL domain containing 2
C3orf62	2.33	0.0123	chromosome 3 open reading frame 62
DDX12P; DDX11	2.3	0.0015	DEAD/H (Asp-Glu-Ala-Asp/His) box polypeptide 12, pseudogene; DEAD/H (Asp-Glu-Ala-Asp/His) box helicase 11
CCHCR1	2.24	0.0014	coiled-coil alpha-helical rod protein 1
TRDC	2.23	0.0432	T-cell receptor delta constant
ALDH2	2.21	0.0432	aldehyde dehydrogenase 2 family (mitochondrial)
SPATA1	2.18	0.0386	spermatogenesis associated 1
CARD6	2.17	0.044	caspase recruitment domain family, member 6
CCDC144CP	2.17	0.0111	coiled-coil domain containing 144C, pseudogene
BMS1P4	2.16	0.0027	BMS1 ribosome biogenesis factor pseudogene 4
FAM120A	2.15	0.0068	family with sequence similarity 120A
SDHAP2; LINC00969	2.14	0.0361	succinate dehydrogenase complex subunit A, flavoprotein pseudogene 2; long intergenic non-protein-coding RNA 969
MIR1299	2.11	0.0403	microRNA 1299
PDCD6	2.08	0.0014	programmed cell death 6
PDK3	2.06	0.0028	pyruvate dehydrogenase kinase, isozyme 3
RAB3D	2.06	0.0037	RAB3D, member of RAS oncogene family
FAM111B	2.02	0.0195	family with sequence similarity 111, member B
IGLL5; IGLV1-36	2	0.0024	immunoglobulin lambda-like polypeptide 5; immunoglobulin lambda variable 1-36
XGY2; XG	2	0.0009	Xg pseudogene, Y-linked 2; Xg blood group
CYP2J2	2	0.0017	cytochrome P450, family 2, subfamily J, polypeptide 2
SNORD14A	−2.01	0.0154	small nucleolar RNA, C/D box 14A
MIR4279	−2.13	0.046	microRNA 4279
MIR3137	−2.17	0.0059	microRNA 3137
HBD	−2.18	0.0494	hemoglobin, delta
MIR514A1	−2.18	0.0399	microRNA 514a-1
IGHD	−2.2	0.016	immunoglobulin heavy constant delta
MMP8	−2.23	0.0204	matrix metallopeptidase 8
LOC101928794	−2.36	0.0249	uncharacterized LOC101928794
SNORD1B	−2.48	0.0488	small nucleolar RNA, C/D box 1B

**Table 4:** DAVID mapping of differentially expressed genes in pGDM versus controls.

Gene List	Enrichment Score *	Cluster
IGHD, TRDC, IGLL5, HBD	2.4	GO:0042571 immunoglobulin complex, circulating
TNFAIP6, F5, CRISPLD2, CST7, CD177, IGHD, TREML5P, MMP8, KIAA1324, ANPEP	0.9	GO:0005615 extracellular space, glycosylation site: N-linked (GlcNAc), glycoprotein
F5, MMP8, ANPEP, PDCD6	0.8	GO:0006508 proteolysis

The functional annotation cluster was obtained using DAVID version 6.8. * The enrichment score is calculated in the log scale of groups of *p*-values in corresponding annotation clusters. The clusters shown here are those with enrichment scores > 0.5.

**Table 5:** Networks with high scores identified through Ingenuity^®^ Pathway Analysis (IPA) in pGDM: The four most impacted networks.

Network Functions	Enrichment Score *	Gene in Network
Cellular Movement, Hematological System Development and Function, Immune Cell Trafficking	17	ANPEP, CYP2J2, CD177, IGHD, TNFAIP6, DYSF, IGLL1, IGLL5
Cell Cycle, Gene Expression, Cellular Development	17	mir-506, PDCD6, PDK3
Cancer, Cell Cycle, Cellular Assembly and Organization	2	ANPEP, mir-506, mir-548, CD177, CST7, PDK3
Cell-To-Cell Signaling and Interaction, Cancer, Gastrointestinal Disease	2	DYSF, F5, CD177, ANPEP, CYP2J2, PDCD6 IGHD, TNFAIP6 ALDH2, C3orf62, mir-506
Hereditary Disorder, Neurological Disease, Organismal Injury and Abnormalities	2	TNFAIP6, ANPEP, F5, HBD, MMP8, ALDH2, PDK3

* The enrichment score is produced by utilizing a *p*-value of less than 0.05.

**Table 6:** List of selected differentially upregulated and downregulated genes in T2D versus controls.

Gene Symbol	Fold Change	*p*-Value	Gene Name
TMTC1	6.75	0.0129	transmembrane and tetratricopeptide repeat containing 1
TRDJ4	5.25	0.0049	T-cell receptor delta joining 4
CLEC12A	4.74	0.0225	C-type lectin domain family 12, member A
DYSF	4.65	0.0013	dysferlin
MT1L; MT1M	4.4	0.0012	metallothionein 1L (gene/pseudogene)
MGAM2	4.38	0.0455	maltase-glucoamylase 2 (putative)
CLEC12B	4.22	0.0174	C-type lectin domain family 12, member B
ANPEP	4.13	0.0008	alanyl (membrane) aminopeptidase
ALPL	4.01	0.0018	alkaline phosphatase, liver/bone/kidney
LOC102724231	3.95	0.0088	uncharacterized LOC102724231
FCGR1A	3.93	0.0289	Fc fragment of IgG, high-affinity Ia, receptor (CD64)
CST7	3.82	0.0004	cystatin F (leukocystatin)
IL3RA	3.58	0.0007	interleukin 3 receptor, alpha (low-affinity)
BMX	3.25	0.0027	BMX non-receptor tyrosine kinase
ADGRG3	3.24	0.0174	adhesion G-protein-coupled receptor G3
ICAM1	3.23	0.0072	intercellular adhesion molecule 1
MIR3939	3.19	0.0242	microRNA 3939
TLR5	3.03	0.0319	Toll-like receptor 5
HAUS4	3	0.0004	HAUS augmin-like complex subunit 4
CASP5	2.99	0.0063	caspase 5
PIK3AP1	2.95	0.0056	phosphoinositide-3-kinase adaptor protein 1
KREMEN1	2.95	0.0104	kringle containing transmembrane protein 1
HCG26	2.94	0.0259	HLA complex group 26 (non-protein-coding)
CARD17	2.91	0.0109	caspase recruitment domain family, member 17
TECPR2	2.89	0.0094	tectonin beta-propeller repeat containing 2
SSH1	2.83	0.0033	slingshot protein phosphatase 1
IL3RA	2.82	0.0005	interleukin 3 receptor, alpha (low-affinity)
TREML2	2.82	0.0069	triggering receptor expressed on myeloid cell-like 2
NQO2	2.8	0.0244	NAD(P)H dehydrogenase, quinone 2
IL1B	2.65	0.0209	interleukin 1 beta
CEP19	2.63	0.0002	centrosomal protein 19kDa
ADGRE1	2.63	0.002	adhesion G-protein-coupled receptor E1
SEMA4A	2.63	0.0178	sema domain, immunoglobulin domain (Ig)
MEFV	2.62	0.0012	Mediterranean fever
SLC25A44	2.62	0.013	solute carrier family 25, member 44
LINC00173	2.61	0.001	long intergenic non-protein-coding RNA 173
BCL6	2.61	0.0068	B-cell CLL/lymphoma 6
SLC26A8	2.61	0.0072	solute carrier family 26 (anion exchanger), member 8
ALDH2	2.61	0.009	aldehyde dehydrogenase 2 family (mitochondrial)
ORAI2	2.59	0.0163	ORAI calcium-release-activated calcium modulator 2
NR6A1	2.58	0.0013	nuclear receptor subfamily 6, group A, member 1
CHSY1	2.58	0.0224	chondroitin sulfate synthase 1
CARD6	2.57	0.0057	caspase recruitment domain family, member 6
LINC01272	2.03	0.0062	long intergenic non-protein-coding RNA 1272
SLC6A6	2.03	0.0096	solute carrier family 6 (neurotransmitter transporter)
MMP9	2.03	0.0129	matrix metallopeptidase 9
MIR133A1; MIR133A1HG	−2.01	0.0193	microRNA 133a-1; MIR133A1 host gene (non-protein-coding)
SNORD9	−2.05	0.0025	small nucleolar RNA, C/D box 9
HK1	−2.07	0.0181	hexokinase 1
TRAJ28	−2.07	0.0419	T-cell receptor alpha joining 28
CUL4A	−2.08	0.0021	cullin 4A
GYPA	−4.36	0.0114	glycophorin A
MMP8	−4.72	0.0214	matrix metallopeptidase 8
AHSP	−5.3	0.0366	alpha hemoglobin stabilizing protein
IFIT1B	−6.9	0.0017	interferon-induced protein with tetratricopeptide repeats 1B

**Table 7:** DAVID mapping of differentially expressed genes in T2D versus controls.

Gene List	Enrichment Score *	GO ID/Cluster
ADGRE1, FFAR2, HCK, MSRB1, TLR5, TLR8, LILRB2, IFIT2, NLRC4, MYD88, TNFSF13B, MEFV, FCGR1A, LILRA4, BCL6, FCGR2A, CLEC4D, EIF2AK2, APOBEC3A_B, TBKBP1, AKIRIN2, SEMA4A	3.7	GO:0045087 innate immune response
CGB1, IER3, OR1A1, NDST1, MMP9, TREML5P, MMP8, GYPA, SIRPB2, CXCR2, ANPEP, TLR5, FCRL6, TLR8, CANT1, SMPDL3A, ITPRIP, HPSE, LTB4R, LILRA4, CSF3R, SEMA3C, SERPINA1, CLEC4D	3.2	GO:0005886 plasma membrane, glycosylation site: N-linked (GlcNAc), glycoprotein
CASP5, TNFRSF1A, IRAK3, NLRC4, MYD88, MEFV, CARD17, NLRP12, CARD6, CASP1 BCL3, BCL6	3.1	IPR011029 death-like domains

The functional annotation cluster was obtained using DAVID version 6.8. * The enrichment score is calculated in the log scale of groups of *p*-values in corresponding annotation clusters. The clusters shown here had enrichment scores > 0.5.

**Table 8:** Networks with high scores identified through IPA in T2D: The five most impacted networks.

Network Functions	Enrichment Score *	Genes in Network
Inflammatory Disease, Inflammatory Response, Cellular Movement	39	MMP8, MMP9, ICAM1, IL1B, IL3RA, IMPDH1, MGAM, mir-21, mir-368
Infectious Diseases, Inflammatory Response, Organismal Injury and Abnormalities	35	BASP1, BMX, BPGM, CACNA1E, CANT1, CARD6, CCR1, CD274, CPD, CSF3R, CSTA, DGAT2, DYSF, ELL, FCAR
Cellular Movement, Hematological System	31	C5AR2, CCR1, CXCR2, DEFA1, MMP9, PLAUR, SEMA4A, SERPINA1, TIMP2, TNFRSF1A
Cell Death and Survival, Cancer	29	BCL6, CD274, FCGR3B, CARD6, ICAM1, IL1B, IL1RN, MYD88, NFIL3, NFKBIA, SIGLEC9, TNFRSF1A, TNFSF13B, MMP9
Connective Tissue Disorders, Immunological Disease	23	MMP25, MMP8, MMP9, NFKBIA, SAMD9L, TIMP2, TLE3, TLR8, TNFRSF1A, TNFSF13B, TRIM21, ZFP36, ZNF281

* The enrichment score was produced by utilizing a *p*-value of less than 0.05.

## Data Availability

All data generated or analyzed during this study are included in the published article.
